# A machine learning model to classify aortic dissection patients in the early diagnosis phase

**DOI:** 10.1038/s41598-019-39066-9

**Published:** 2019-02-25

**Authors:** Da Huo, Bo Kou, Zhili Zhou, Ming Lv

**Affiliations:** 1 School of Management, Xi’an Jiaotong University, Xi’an, 710049 China; 2 Department of System Engineering and Engineering Management, City University of Hong Kong, Kowloon Tong, Hong Kong SAR; 3 Department of Otorhinolaryngology-Head & Neck Surgery, The First Affiliated Hospital, Medical School of Xi’an Jiaotong University, Xi’an, 710061 China; 4 State Key Laboratory for Manufacturing Systems Engineering, Xi’an Jiaotong University, Xi’an, 710049 China

## Abstract

Aortic dissection is one of the most clinical-challenging and life-threatening cardiovascular diseases associated with high morbidity and mortality. Aortic dissection requires fast diagnosis and timely therapy. Any delay or misdiagnosis can cause severe consequence to aortic dissection patients with even higher mortality. To better help physicians identify the potential dissection within the scope of all misdiagnosed patients, this paper describes a method which is developed with data mining methods for aortic dissection patient classification and prediction in the phase of early diagnosis. Various machine learning algorithms were used to build the models which were all trained and tested on the patient dataset with cross validation. Among them, Bayesian Network model achieved the best performance by predicting at a precision rate of 84.55% with Area Under the Curve (AUC) value of 0.857. On this basis, the Bayesian Network model can help physicians better with early diagnosis of aortic dissection in clinical practice. Beyond this study, more data from diverse regions and the internal pathology can be crucial to further build a universal model with broader predictive power.

## Introduction

Cardiovascular diseases are among the major causes of death in most developed countries and many if not all developing countries^1^. Among all the categories of cardiovascular diseases, aortic dissection is fatal with high mortality. The aorta is a complex organ, of which the wall has a 3-layered anatomic configuration. When aortic dissection occurs, blood flows between the layers of the aortic walls then forces the layers apart and creates a new secondary channel^2^. Previous literature examined the aortic dissection patients and found some features of the population. For example, a typical aortic dissection patient is a gentleman in his 60 s or 70 s with hypertension, prior cardiac surgery, bicuspid aortic valve, and a history of Marfan syndrome^3^. However, these features are extracted by only examining the confirmed patients without comparing to the features of other easy-to confuse diseases. Some works from the previous literature show that patients with aortic dissection share pathologic similarities with other diseases^4–6^. which may not be so useful to distinguish the real ones from all misdiagnosed cases.

In clinical practice, aortic dissection patients usually arrive in an emergency with the common abrupt onset of severe chest pain, which makes it critical and challenging to successfully predict and identify aortic dissection as the actual cause out of all the possible ones^7^. Up to 30% of aortic dissection patients are initially misdiagnosed to have other conditions, such as acute coronary syndromes, non-dissecting aneurysms, pulmonary embolism, or aortic stenosis^8–10^. Until now, no unique symptoms have been found for aortic dissection and it always requires imaging detection like chest X-ray and ECG (Electrocardiograph) to further confirm. Acquiring such detection is time-consuming that is not ideal since speed is of utmost importance of aortic dissection diagnosis. However, such tests for misdiagnosed cases can be crucial for the coming therapy after confirmation which may differ in terms of other conditions^11^. Since there is a wide range of clinical symptoms for aortic dissection patients, misdiagnosed cases should go through quick risk stratification and management^12^. Beyond traditional detection schema of processing ECG and chest X-ray which is slow that may delay the timely therapy, a new method that can assist physicians to fast and reliably identify the aortic dissection patients from all misdiagnosed cases is badly in need.

In order to fast and reliably identify, or in other words, to classify patients into aortic dissection category if they are, it is necessary to conduct methods which have high effectiveness and efficiency in classifying. In view of this strong demand in clinical practice, machine learning classification can serve as one solution. It is a supervised machine learning technique that assigns items to target classes when class labels are known^13^. Classification, together with other machine learning techniques, has a potential to make a huge impact on healthcare and biomedical domain^14^. In several works, classification approach enhanced by attribute selection techniques has been successfully applied in medical informatics^15–18^. Nonetheless, few researchers focus on the early diagnosis and prediction of aortic dissection due to the imbalanced geographical distribution of patients which makes it difficult to collect high-volume and high-quality dataset to proceed such method.

In our study, we proposed a prediction model by applying classification analysis to classify aortic dissection positive patients from all misdiagnosed cases in the phase of early diagnosis. Such model can help physicians to fast spot aortic dissection positive patients out of all misdiagnosed cases, and to lose no time in understanding the situation and estimating the progression after admission. After training on the real-case data, this prediction model can set the priority of patients by classifying them into two group. The predicted aortic dissection positive patients need more eyes on whereas the predicted aortic dissection negative patients are more likely to be eventually diagnosed with other diseases. In the past, the decisions made in the early diagnosis phase are primarily based on physicians’ expertise and consultation, which can be a barrier to a successful diagnosis. The new model reduces this barrier since all the required data come from admission information plus several simple and routine tests. It saves both patients’ time and hospital resources by making it possible to provide the timely therapy for the most needed patients.

## Results

### Attributes selection

We examined both the confirmed and misdiagnosed aortic dissection cases in the past three years to build the initial dataset. The raw data may contain irrelevant or redundant information that can make knowledge discovery more difficult. Attributes in this dataset include personal information details, family status, career information, drug allergies, and medical test results. For the aim of quick classification in early diagnosis phrase, the data need to be easy to acquire and have the lowest dimensions while maintaining the accuracy.

The attribute selection process contains two steps: hand selection then algorithm evaluation.

In the first step, we eliminated attributes of highly personal details, general administrative records and specific characteristics which do not apply to all patients. The remaining 40 attributes after the first step include parts of EMRs, blood routine test results and lipid profile: SEX, AGE, OCCUPATION, MARITAL STATUS, IN_TYPE (patient admission type), WEATHER, TEMPRETURE (both highest and lowest), IN_DEPT (first admission department), CHOI, TG, Lp(a), HDL-C, LDL-C, APOA, APOB, APOE, WBC, #BASO, BASO%, #EO, EO%, HCT, HGB, #LYMPH, LYMPH%, MCH, MCHC, MCV, #MONO, MONO%, MPV, #NEUT, NEUT%, PCT, PDW, P-LCR, PLT, RBC, RDW-CV, RDW-SD.

Then in the second step, we applied CFS subset evaluation method with the greedy algorithm on these attributes. CFS method selects the following thirteen attributes that have the best predictive ability with lowest degree of redundancy: IN_TYPE (Admission Type), IN_DEPT (First Admission Department), CHOL (Total Cholesterol), TG (Triglyceride), Lp(a) (Lipoprotein a), HDL-C (High-density Lipoprotein), APOB (Apolipoprotein B), APOE (Apolipoprotein E), WBC (White Blood Cell Count), LYMPH% (Lymphocyte Percentage), #MONO (Monocyte Count), RBC (Red Blood Cell Count) and RDW-CV (Red Cell Distribution Width, Coefficient of Variation). These thirteen attributes would be used to build the machine learning classification model and for future clinical prediction during the early diagnosis phase. These thirteen attributes would be used to build the machine learning classification model and for future clinical prediction during the early diagnosis phase.

### Basic Characteristics of the patients

**Figure 1:**
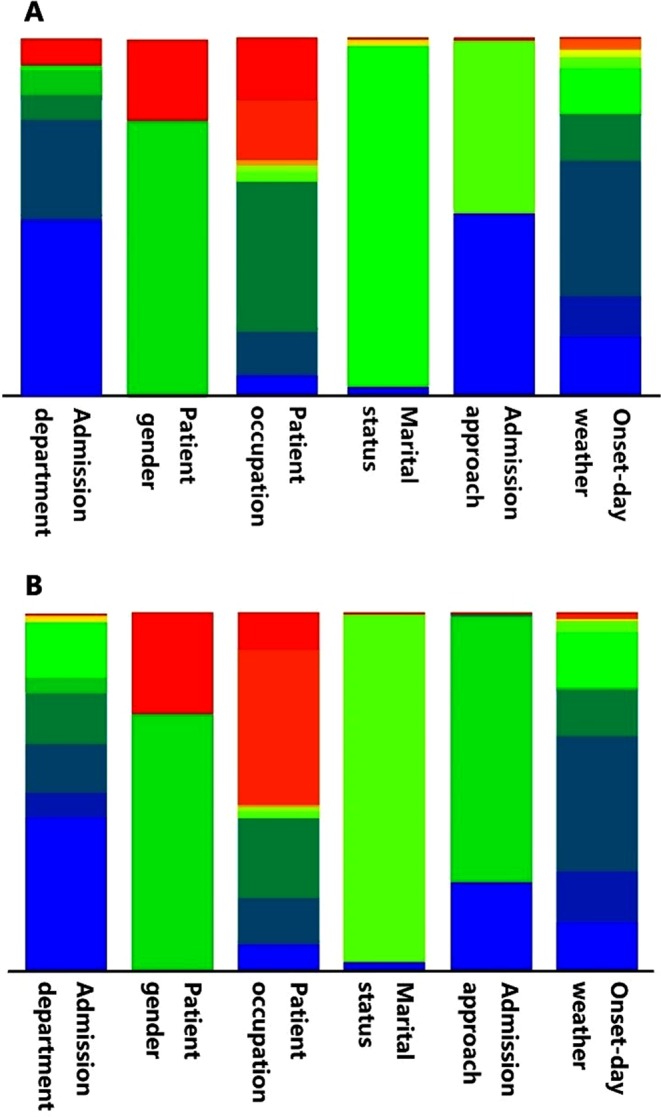
Stacked bar chart of selected patient nominal attributes. (**A**) Nominal attributes of aortic dissection positive patients; (**B**) nominal attributes of aortic dissection negative patients. Different colours in one column represent the possible nominal values only in that particular attribute. Same colour across different columns has no necessary connection or implying same value interval.

**Figure 2:**
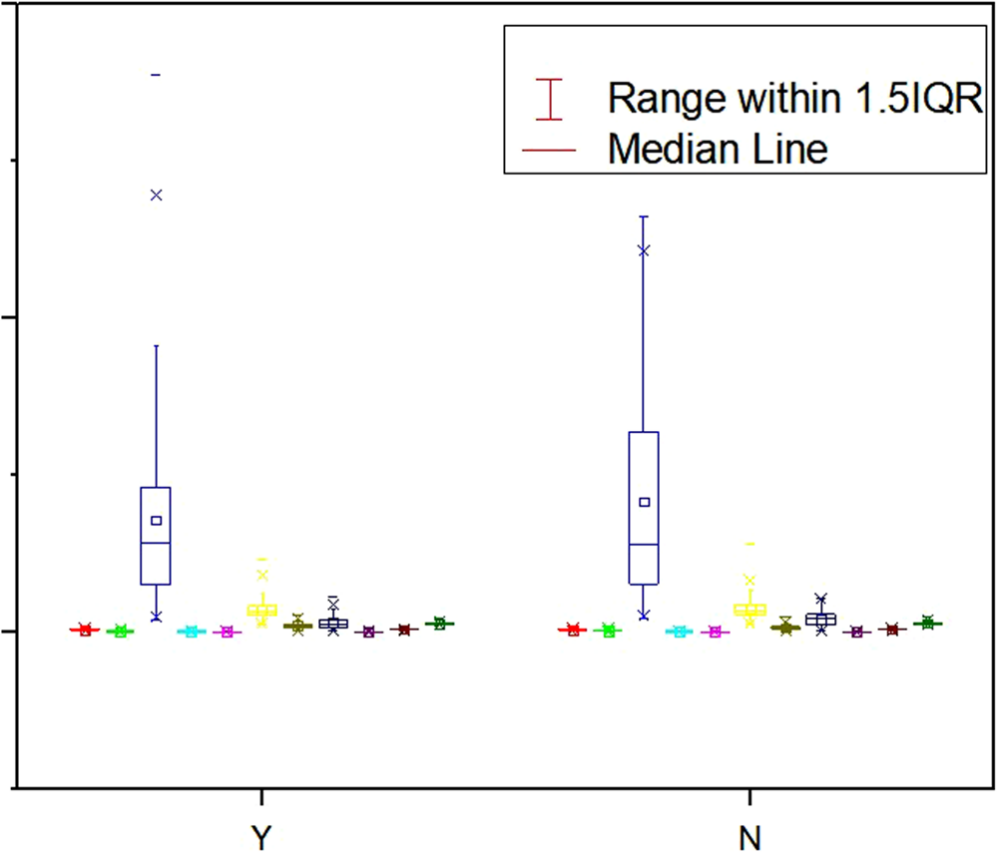
Grouped box chart of selected patient numerical attributes. Y: aortic dissection positive patients. N: aortic dissection negative patients. For each group, the boxes are in the order of CHOL, TG, Lp(a), HDL-C, APOB, APOE, WBC, LYMPH%, #MONO, RBC and RDW-CV from left to right.

492 cases including 372 male patients and 120 female patients were recruited in the study. They are either with aortic dissection or misdiagnosed as aortic dissection by physicians in early diagnosis phase. The descriptive statistical analysis was performed to get the perceptual image of our case dataset and to show the basic characteristics of the patients. The analysis of selected attributes after applying the attribute selection algorithm with some other descriptive attributes including age, gender, marital situation, occupation, and weather condition, were presented by figure or table based on their nominal or numeric nature. Nominal attributes of aortic dissection positive patients were shown in a stacked bar chart (Fig. 1). The colour set in one attributes column represents the possible nominal values in that specific attribute; the area size of each colour is relative to how many instances an attribute has for that specific nominal value. Same colour across different bars has no necessary connection or implying same value interval. The specific values of each attribute are given in Table 1. Through Fig. 1 and Table 1, it is difficult to tell the overall difference between aortic dissection patients and the misdiagnosed patients. A grouped box chart analysis of selected numeric attributes is shown in Fig. 2. There is no distinction between aortic dissection patients and misdiagnosed patients among all attributes except Lp(a) of misdiagnosed patients has a wide range of distribution. Descriptive analysis of numeric attributes was presented in (Table 2).

**Table 1:** Patients characteristics with selected nominal attributes.

Attributes	No. Positive	% Positive	No. Negative	% Negative
**Gender**				
Male	255	77.3%	116	71.6%
Female	75	22.7%	46	28.4%
**Marital Status**				
Unmarried	8	2.4%	4	2.5%
Married	314	95.2%	157	96.9%
Widowed	6	1.8%	1	0.6%
Divorced	2	0.6%	0	0%
**Admission Approach**				
Emergency	168	50.9%	40	24.7%
Outpatient	159	48.2%	120	74.1%
Transferred-in	3	0.9%	2	1.2%

**Table 2:** Patients characteristics with selected numeric attributes. Each attribute is showed separately by the class label of aortic dissection positive or aortic dissection negative. P: aortic dissection positive; N: aortic dissection negative. The onset-day air temperature interval and weather condition which shows in (figure 1), was initially involved to see if there was a high connection between the natural environment and an aortic dissection attack.

Variables		Minimum	Maximum	Mean	STD
**Clinical Data**					
Age, year	P	20	87	54.0	13.1
	N	21	87	57.6	14.4
Male, n (%)	P	N/A	N/A	255 (77.04%)	N/A
	N	N/A	N/A	116 (71.60%)	N/A
Low Air Temp, °C	P	−12	28	9.3	9.2
	N	−5	27	12.3	8.8
High Air Temp, °C	P	−2	37	18.4	9.9
	N	1	38	21.3	9.8
**Lipid Profile**					
CHOI, mmol/L	P	1.95	7.34	3.46	0.83
	N	2.14	7.59	3.57	1.02
TG, mmol/L	P	0.25	7.40	1.28	0.77
	N	0.40	8.84	1.53	0.95
Lp(a), mg/L	P	20.00	887.30	166.67	127.01
	N	20.60	660.00	194.41	144.66
HDL-C, mmol/L	P	0.32	1.95	0.97	0.27
	N	0.43	1.92	0.90	0.23
APOB, g/L	P	0.28	1.55	0.67	0.18
	N	0.32	1.50	0.71	0.23
APOE, mg/L	P	11.00	115.50	33.39	12.56
	N	13.20	139.80	34.99	14.30
**Blood Routine Examination**					
WBC, 10^9^/L	P	2.05	26.16	10.12	4.14
	N	2.00	24.14	7.24	3.08
LYMPH%, %	P	2.00	57.10	14.89	9.38
	N	1.80	54.51	21.09	11.12
MONO#, 10^9^/L	P	0.00	4.60	0.62	0.41
	N	1.83	6.22	4.05	0.83
RBC, 10^12^/L	P	2.38	6.08	4.17	0.71
	N	0.01	1.50	0.43	0.23
RDW-CV, %	P	11.40	21.80	13.37	1.11
	N	11.70	23.00	13.87	1.60

### Classification models

We used Weka toolkit to employ four common classifiers on our dataset including Bayesian Network, Naïve Bayes, J48, and SMO. Grid search algorithm was used to find the optimal parameters for each model thus could achieve best possible performance^19^. The result of ZeroR classification and prediction rule was set as the baseline to which, we compared the performance of each model to the accuracy of physicians which is set as the benchmark *r*_0_.

1 $${r}_{0}=\frac{\#positive\_cases}{\#positive\_cases+\#negative\_cases}$$

**Figure 3:**
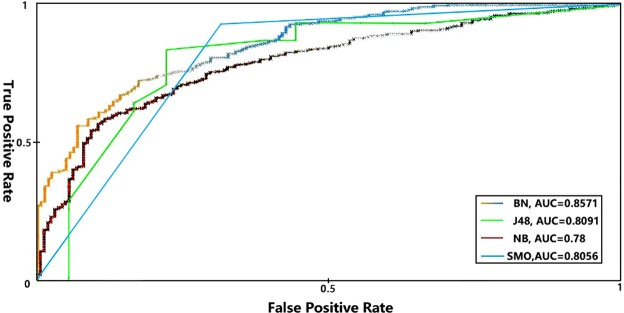
ROC (Receiver Operating Characteristic) curves show the performance of models using different machine learning algorithms. BN: Bayesian Network; NB: Naïve Bayes; J48: Java implementation of the C4.5 decision tree algorithm; SMO: Sequential Minimal Optimization. Bayesian Network classifier achieved the maximum Area Under ROC Curve (AUC) as 0.8571.

The benchmark prediction precision is 67.07% with neutral AUC value 0.4951. Bayesian Network model achieved precision rate as 84.55% with AUC value 0.8571. Naïve Bayes model achieved precision rate as 79.88% with AUC value 0.78. J48 model achieved precision rats as 76.14% with AUC value 0.8091. SMO model achieved precision rate as 82.11% with AUC value 0.8056. The ROC curves are shown in Fig. 3. Bayes Net model has the best performance among four classifiers. The details of each model can be found as online supplementary materials.

### Performance on emergent patients

Even though our dataset contains more nonurgent patients than emergent patients (284:208). Emergent patients usually have more critical situations which is more likely to cause patient death. For this reason, we need to pay extra attention to emergent patients. We reconduct the process and find our model can work even better on emergent patients. The speed of early diagnosis is even more crucial for emergent patients. The CFS attribute selection method select 4 of 40 attributes with the highest performance (IN_DEPT, APOB, WBC, #MONO). This can significantly reduce the dimension of required data and can help physicians handle the situation better. The performance of each selection method is summarized in Table 3.

**Table 3:** Model performance on emergent patients.

Classifier	Precision	AUC
Benchmark	80.77%	0.4952
Bayesian Network	86.54%	0.8138
Naïve Bayes	86.53%	0.8057
SMO	84.13%	0.6256
J48	81.73%	0.5905

### Contributions to clinical diagnosis

One of our research purposes is to find the inherent connection between the multiple attributes of the aortic dissection patients and then to extract a model that can predict the disease better than previous physician judgement. The pathogenesis of aortic dissection is so complex with diverse patient symptoms. Physicians relying on previous experience often leads to misdiagnosis and therefore fails to provide timely treatment. Due to the low morbidity rate and high mortality rate of Aortic dissection, physicians in the past did not have enough cases to summarise the characteristics of such disease, let alone somehow develop an efficient and reliable model for prediction and early diagnosis. Our study involved 527 cases of both confirmed and misdiagnosed aortic dissection patients. We presented a model that can correctly predict the aortic dissection at the ratio over 85%. Compared to the 67% baseline, we improved the prediction precision which is critical to provide appropriate treatment including real-time monitoring for early diagnosis.

For the misdiagnosed cases, we find that many of them are simply hypertension patients (22 cases), coronary artery patients (15 cases), unstable angina pectoris patients (14 cases), chronic nephritis patients (11 cases). To better distinguish these diseases with aortic dissection can be our focus in future research.

## Discussion

Machine learning classification, unlike clustering that is another branch of machine learning, is a supervised learning technique which is built on the previous knowledge of raw dataset and relevant class labels^20^. Since the dataset in this study is the final record version, each case has specific and clear label besides all the relevant attributes. We fully utilized this complete dataset for knowledge mining by machine learning classification approach. Along with classification, we proceeded attribute selection in pre-processing step to remove irrelevant and redundant attributes that can be noises when processing classification. Theoretically, the model we proposed can help classify aortic dissection positive patients apart from other misdiagnosed cases. The further work may include integrating this model and algorithm into the current system or developing an independent application with data imported from EMR system.

To help us better understand the nature of our dataset and the prediction model, we tried to analyse the characteristics of the selected attributes. The selected thirteen attributes contain six from lipid profile and five from blood routine examination while the other two are patient admission attributes. That 6 of total 8 lipid-profile test attributes were used to build our model indicates the lipid profile can be very crucial to the clinical prediction in early diagnosis phase. Previous research finds that many aortic dissection patients show hyperlipidemia, especially for male patients^21,22^. Lipids infiltrating the aortic wall can cause an immune response known as inflammatory aortic aneurysm which weakens the aortic wall, hastens the wall expansion and causes higher wall stress^23^. Our model and analysis show that aortic dissection has no distinct gender or occupation trend compared to.

In conclusion, our proposed risk-stratification model can help physicians with early classification and prediction of aortic dissection patients from all the misdiagnosed cases, which can save test and diagnosis time by providing timely therapy for the most urgent patients. Besides, the thirteen attributes after attribute selection process can give physicians new inspiration and reference for further understanding aortic dissection and deeper expending the relevant pathogenesis knowledge. However, the prediction model is based on the misdiagnosed cases that come from a regional medical centre in North-western China. The geographical distinction may lead to a varied model when applying to patients from other regions or countries. Also, more knowledge of inherent pathogenesis is always needed whether for a better understanding and therapy of the disease or for a more precise model with more representative attributes.

## Methods

### Study population

There were 526 cases of both confirmed and misdiagnosed aortic dissection involved in our study from May 2013 to November 2016. In 34 cases, patients died shortly after admission and lacked chemical test data like blood routine examination and lipid profile. These 34 cases were excluded from the study population thus we have a population size of 492. All data except weather condition and air temperature were collected by hospital’s EMR system. Weather and temperature data were collected from tianqi.com which is a public weather information service provider. All patients were admitted to the First Affiliated Hospital of Xi’an Jiaotong University either in emergency or outpatient approach. After diagnosed in the first admitted department if not cardiac, they were transferred to the cardiac department because of the diagnosis or suspicion of aortic dissection. All patients admitted if not died earlier had accepted blood routine examination and lipid profile test. These chemical test data and the data previously mentioned were used for data mining.

### Attribute selection

By using attribute selection techniques, we select a subset of attributes which are most relevant for classification. CFS subset evaluation was employed in our study for evaluating the contribution of attribute subsets to find the relevant attributes^24^. It is the first of the methods that evaluate subsets of attributes rather than individual attributes^25^. For the search method, a forward greedy search was applied to search through the space of attribute subsets. CFS algorithm involves a heuristic (1) that considers the usefulness of each feature for predicting the class with the level of intercorrelation among them.

2 $$Meri{t}_{s}=\frac{k\overline{{r}_{cf}}}{\sqrt{k+k(k-1)\overline{{r}_{ff}}}}$$

*Merit*_*s*_: the heuristic “merit” of a feature subset *S* containing *k* features, $\overline{{r}_{cf}}$: the average feature-class correlation, $\overline{{r}_{ff}}$: the average feature-feature intercorrelation.

### Cross validation

The previous study proved that using the whole dataset for training may not fully discover the nature of the dataset thus fail to build the reliable classification model for further prediction. To better train our model out of the dataset and build it more robust, we used a tenfold cross validation technique which divides the dataset equally into 10 folds and iterates with nine folds for training and the rest one for testing until each fold is used for testing. The previous study evidently showed and theoretically backed up that 10 is the right number of folds to get the best error estimate^26^.

### Machine learning classification

The Weka machine learning toolkit was used for building the classification model. Before processing the dataset, we applied attribute selection techniques to select critically relevant attributes out of the overall attributes in EMR system. The selected attributes helped us to better discover the nature of the patient dataset as well as build a more efficient prediction model. When processing, we used multiple classifiers including SMO, J48, Naïve Bayes, Bayesian Network. The Naïve Bayes classifier assumes that all attributes are conditionally independent with given class label. By maximum likelihood estimation, the prior probability of each class label and the conditional likelihood of each attribute value with given class label are learned from the training dataset. After learning process, the trained classifier assigns a class label to the instance in the test set, which maximises the likelihood function. Unlike Naïve Bayes approach, Bayesian Network encodes the set of conditional independence assumption into itself. Besides, the condition is not only on the class label but on any other parents of the given attribute as well. Finally, we assign the instance to that class label which has the highest posterior probability. J48 is an open source Java implementation of the C4.5 decision tree algorithm which is an extension of the ID3 algorithm with some improvements. Two of these improvements is the ability to handle both continuous and discrete attributes and to handle training dataset with missing values. These two improvements are crucial in our study because the aortic dissection dataset has some mixed nominal and numeric attributes with some missing values. Like some other improved Support Vector Machine (SVM) algorithm, SMO is an iterative algorithm which breaks the problem into a series of sub-problems. The only difference is SMO breaks the problem to the smallest scale. A unique feature of SMO is that it solves the problem analytically thus avoids the complex iterations in traditional SVM algorithm which saves computing time and memory and excludes the errors accumulated from iterations.

### Model evaluation

Model evaluation is the key to knowledge representation and to making a practical impact from model to the real world. For our study, which is a typical classification problem, it is natural to use the error rate or precision rate to measure a classifier’s performance. The selected classifier predicts the class of each instance. The precision rate is the proportion of successes made by a classifier over the whole instance set. ROC curves were also used to evaluate the model performance which can overcome the distribution bias of class labels. ROC curves plot the true positive (TP) rate on the vertical axis against the true negative (TN) rate on the horizontal axis.

3 $$Precision\,Rate=\frac{TP+TN}{TP+TN+FP+FN}\times 100{\rm{ \% }}$$

4 $$TP\,Rate=\frac{TP}{TP+FN}\times 100 \% $$

5 $$FP\,Rate=\frac{FP}{FP+TN}\times 100 \% $$

*TP*: True Positive, *FP*: False Positive, *TN*: True Negative, *FN*: False Negative.

All sensitive data relative to personal information were filtered out from the study. All data collection and the study protocol were approved by the ethics committee of the First Affiliated Hospital, Xi’an Jiaotong University, in compliance with the Declaration of Helsinki. All the participants were informed of the nature and purpose of the study and signed written informed consent.

## Data Availability

The masked data can be provided for reconduct and discussion upon request.
